# Sex-Based Differences in Systemic Sclerosis Among Egyptian Patients: Insights from a Multicenter Observational Study in a Genetically Distinct North African Mediterranean Population

**DOI:** 10.3390/jcm14217574

**Published:** 2025-10-25

**Authors:** Hesham Hamoud, Mahmoud Ibrahim Risha, Mohamed Elsayed Hanafy, Abdelhfeez Moshrif, Rasha A. Abdel-Magied, Gihan Omar, Adel M. Elsayed, Abdelazeim Elhefny, Sameh Mobasher, Mervat Abo Gabal, Manal Hassanien, Fatma H. EI Nouby, Khaled A. A. Abdelgalil, Giulio Forte, Luca Navarini, Onorina Berardicurti, Piero Ruscitti, Francesco Ciccia, Roberto Giacomelli, Vasiliki Liakouli

**Affiliations:** 1 Department of Rheumatology, University of Al-Azhar, Cairo 11651, Egypt; hamoud.hesham@yahoo.com (H.H.); dr.m7risha@yahoo.com (M.I.R.); drhanafy2019@gmail.com (M.E.H.); drmoshrif@azhar.edu.eg (A.M.); drmervat40@yahoo.it (M.A.G.); 2 Rheumatology and Rehabilitation Department, Faculty of Medicine, Minia University, Minya 61511, Egypt; rasha_ali@mu.edu.eg (R.A.A.-M.); gihan_omar@mu.edu.eg (G.O.); 3 Department of Rheumatology, University of Ain Shams, Cairo 11566, Egypt; adel.mahmoud@med.asu.edu.eg (A.M.E.); elhefny@med.asu.edu.eg (A.E.); drsom7@hotmail.com (S.M.); 4 Department of Physical Medicine, Rheumatology and Rehabilitation, University of Assuit, Asyut 31527, Egypt; manal_hassanien@yahoo.com (M.H.); fatmanoby@med.aun.edu.eg (F.H.E.N.); 5 Department of Rheumatology and Physical Medicine, University of Aswan, Tingar 81528, Egypt; khaled.rheuma@gmail.com; 6 Rheumatology Unit, Department of Precision Medicine, University of Campania “Luigi Vanvitelli”, 80131 Naples, Italy; forgiulio97@gmail.com (G.F.); francesco.ciccia@unicampania.it (F.C.); 7 Clinical Unit of Immunorheumatology, Fondazione Policlinico Universitario Campus Bio-Medico, Via Álvaro del Portillo, 200, 00128 Roma, Italy; l.navarini@unicampus.it (L.N.); o.berardicurti@policlinicocampus.it (O.B.); r.giacomelli@unicampus.it (R.G.); 8 Research Unit of Immunorheumatology, Department of Medicine and Surgery, Università Campus Bio-Medico di Roma, Via Alvaro del Portillo, 200, 00128 Roma, Italy; 9 Department of Biotechnological and Applied Clinical Sciences, University of L’Aquila, 67100 L’Aquila, Italy; piero.ruscitti@univaq.it

**Keywords:** sex differences, EScSG, Medsger severity scale, modified Rodnan skin score, multicenter study, systemic sclerosis

## Abstract

**Background:** Systemic Sclerosis (SSc) is a heterogeneous autoimmune disease with variable clinical expression influenced by genetic, environmental, and sex-related factors. Understanding sex-based differences in disease phenotypes and severity can improve personalized management strategies, especially in underrepresented populations. This study aims to explore sex-based differences in disease phenotypes and severity in a population with a distinct genetic background. **Materials and Methods:** This cross-sectional study included 197 SSc patients (177 females and 20 males) enrolled from 5 tertiary care centres across Egypt. All participants met the 2013 ACR/EULAR classification criteria for SSc and the criteria proposed by LeRoy and Medsger. The demographic, clinical, and serological data were collected and defined according to the previously developed severity score and activity index. **Results:** This study highlights key sex-related differences in disease severity and management. Egyptian male patients exhibited more severe skin involvement and were more likely to receive more aggressive treatment regimens, including corticosteroids and phosphodiesterase inhibitors. Conversely, female patients demonstrated a higher frequency of moderate general systemic involvement and comparatively lower rates of pulmonary complications. **Conclusions:** Sex-related differences in Egyptian SSc patients appear limited, suggesting that population-specific genetic and environmental factors may play a more prominent role in disease expression than sex alone.

## 1. Introduction

Systemic sclerosis (SSc), or scleroderma, is a rare autoimmune connective tissue disorder marked by widespread microvascular damage, immune dysregulation, and progressive fibrosis of the skin and internal organs. Clinically, SSc is categorized into two main subtypes based on the extent of skin involvement: diffuse cutaneous SSc (dcSSc) and limited cutaneous SSc (lcSSc) [1,2]. A third subset, known as SSc sine scleroderma, includes patients with internal organ involvement and SSc-specific autoantibodies but without cutaneous fibrosis [3].

Although SSc can affect individuals of any sex, growing evidence indicates that sex significantly influences disease susceptibility, clinical expression, and outcomes. Women are more frequently diagnosed with SSc; however, men often exhibit more severe disease manifestations and worse prognoses [4]. Despite this, the extent and nature of sex-related differences remain incompletely understood, particularly within specific regional or ethnic populations.

SSc is also notable for its substantial clinical heterogeneity. Factors such as genetic background, environmental exposures, and healthcare disparities contribute to the variability in disease presentation and progression. Comparative analyses of genetically diverse yet geographically close populations have proven instrumental in unraveling these complex interactions. In this context, the emerging discipline of gender medicine offers a valuable framework to explore how biological sex and gender-related sociocultural roles shape disease development, diagnosis, and treatment.

Gender medicine is an interdisciplinary field that investigates how sex-based biology and gender identity affect health, disease progression, and treatment responses. It also considers how societal roles, behavior patterns, and access to healthcare systems impact health outcomes [5]. By addressing these variables, gender medicine aims to refine diagnostic accuracy, personalize therapeutic strategies, and ultimately improve health outcomes across populations.

Egyptian patients represent a particularly informative group in which to explore these dimensions. The interplay between genetic predisposition, environmental influences, healthcare infrastructure, and cultural norms may uniquely influence the clinical profile and disease burden of SSc in this population [6]. Preliminary monocentric retrospective data suggest that male Egyptian patients with SSc may experience more aggressive disease [7], but high-quality, multicenter studies on sex-specific differences in this setting are still lacking.

This study aims to investigate sex-related differences in disease phenotype, serological characteristics, clinical outcomes, and treatment approaches in a multicenter cohort of Egyptian patients with SSc. By integrating regional and sex-based analyses, this research seeks to enhance our understanding of SSc heterogeneity and support the development of more tailored and equitable care strategies.

## 2. Materials and Methods

### Study Design, Patients, and Assessment of Disease Characteristics

The study population included 197 SSc patients from 5 tertiary Rheumatologic Units, throughout the whole Egypt with a high experience in the management of this disease. This was a retrospective, cross-sectional study, and all patients fulfilled the American College of Rheumatology/European League Against Rheumatism (ACR/EULAR) 2013 classification criteria [8] and criteria proposed by LeRoy and Medsger [9] were consecutively enrolled from 1 January 2017, to 31 December 2017. The cross-sectional demographic, clinical, serological data, and treatments were collected and defined according to the previously developed severity score [10] and activity index [11]. This sub-study is part of a previously approved study by the Ethical Committee of Azienda Sanitaria Locale 1 Avezzano-Sulmona-L’Aquila, Italy (Protocol No. 0151147/20) [6], which also included Italian participants. The sub-study was conducted in accordance with Good Clinical Practice guidelines and the Declaration of Helsinki. All patients gave their written consent after clearly explaining the study objectives and procedures.

All patients were subjected to full history taking, clinical examination, and relevant laboratory and radiological investigations related to their SSc. Clinical, serological, and radiological disease-related features included disease duration from the onset of the first non-Raynaud’s phenomenon (RP), disease subset according to LeRoy criteria [9], anti-nuclear antibodies (ANA) and SSc-specific autoantibodies, ischemic digital ulcers, modified Rodnan skin score (mRSS), interstitial lung disease (ILD) assessed by high-resolution computed tomography (HRCT), and pulmonary arterial hypertension (PAH) measured by echocardiography. Patients with estimated systolic pulmonary arterial pressure (sPAP) > 35 mmHg are at higher risk of presenting PH with the majority of SSc studies investigating PAH screening used sPAP as a suitable parameter for this goal [12]. The European Scleroderma Study Group (EScSG) activity index was used to assess disease activity [13,14]. Disease severity was assessed by the core set of variables proposed by Medsger et al. [15] with percentage cut-offs determined according to the Medsger severity scale, which classifies disease severity into five categories: 0 (normal), 1 (mild), 2 (moderate), 3 (severe), and 4 (end-stage). The current therapy was also recorded.

While the cohort included patients from multiple centers, all participating rheumatology units adhered to a shared data collection protocol based on predefined clinical definitions and standardized assessment tools. Prior to data entry, clinicians received joint training on variable definitions and scoring systems to ensure consistency across sites. Furthermore, data were reviewed centrally for completeness and consistency, and any discrepancies were resolved through direct communication with the respective centers.

## 3. Statistical Analysis

Data were collected and analysed using the statistical package SPSS version 20 (SPSS Inc., Chicago, IL, USA). The distribution of each continuous variable was assessed for normality prior to analysis. Normally distributed continuous variables were expressed as mean  ±  standard deviation (SD), while non-normally distributed variables were expressed as median and interquartile range (IQR). Categorical variables were presented as absolute numbers and percentages. Comparisons of continuous variables between females and males were conducted using the Student’s *t*-test for normally distributed data or the Mann–Whitney U test for non-normally distributed data, as appropriate. Pearson’s chi-square test or Fisher’s exact test was used to compare the frequency distribution of categorical variables between males and females. We performed an exploratory univariable logistic regression analysis to evaluate the association between each covariate of the Medsger severity scale and sex. However, due to the small number of male patients in several Medsger severity score strata, multivariable logistic regression analysis could not be performed reliably. Correlations between continuous variables and mRSS score were assessed using Spearman’s rank correlation. Furthermore, we performed univariable and multivariable linear regression analyses to identify clinical factors associated with the extent of skin involvement, using mRSS as a continuous outcome. Lastly, to reduce the imbalance between sexes, a matched sub-analysis was conducted, pairing males and females by disease severity to better assess sex-related differences. Statistical significance was set at *p* < 0.05.

## 4. Results

### 4.1. Demographic and Disease-Related Features of SSc Patients by Sex

Among 197 patients with SSc enrolled from 5 tertiary centres across Egypt, a marked predominance of females was observed (89.8%, n = 177) compared to males (10.2%, n = 20). The mean age at disease onset was comparable between sexes (41.2  ±  12.5 in females vs. 39.1  ±  13.1 years in males; *p* = 0.424), and disease duration from the first non-Raynaud’s symptom did not differ significantly (7.5  ±  6.1 vs. 5.6  ±  3.0 years; *p* = 0.182).

Analysis of disease subsets revealed that limited cutaneous SSc (lcSSc) was the predominant phenotype in both sexes, affecting 59.9% of females and 70% of males, while diffuse cutaneous SSc (dcSSc) was seen in 40.1% of females and 30% of males (*p* = 0.308). Regarding immunological features, a high prevalence of ANA positivity was found in both groups (93% of females, 91.3% of males), and similar rates of anticentromere (ACA) and anti-topoisomerase I (ATA) antibodies were recorded, with no significant sex-based difference. Clinical manifestations such as skin involvement, estimated via the modified Rodnan skin score (mRSS ≥ 14), were substantial and similar between groups (79.8% of females vs. 73.7% of males; *p* = 0.535). Frequencies of elevated systolic pulmonary artery pressure (sPAP > 35 mmHg), interstitial lung disease (ILD), history of digital ulcers, and active disease (EScSG activity index) showed no significant sex differences. However, corticosteroid therapy was more frequently administered to male patients (55% vs. 30.5%; *p* = 0.027), and there was a trend towards greater use of phosphodiesterase inhibitors among males (25% vs. 10.2%; *p* = 0.050). No sex differences were found in the use of immunosuppressive therapies. A comprehensive summary of demographic, immunological, and clinical data by sex is provided in Table 1. Finally, a matched sub-analysis was conducted to reduce sex imbalance by pairing males and females according to age and disease severity; however, the findings were not statistically significant and should therefore be interpreted with caution (Supplementary Table S1).

#### Differences in the Severity of Organ Involvement by Sex

Key sex differences emerged in the severity of organ involvement, as assessed by the Medsger severity scale. Notably, in the descriptive analysis, male patients demonstrated significantly greater severity of skin involvement, with 15.5% exhibiting severe skin disease compared to only 4.5% of female patients (*p* < 0.044). In contrast, general manifestations were more prominent in females, with 25.6% presenting with moderate general organ involvement, whereas none of the male patients showed involvement in this domain (*p* = 0.010). Pulmonary involvement represented another domain of marked sex disparity. A significantly lower proportion of females (48.6%) exhibited no lung involvement compared to males (80%) (*p* < 0.008). Furthermore, moderate to severe pulmonary involvement was reported in 12.4% of female patients, while no male patients showed severe lung disease (*p* = 0.094). Importantly, ILD in Table 1 was defined radiologically by HRCT, while pulmonary involvement in the Medsger’s severity score (Table 2) reflects functional impairment. Thus, patients with ILD on HRCT but preserved lung function could still be classified as ‘0’, explaining the apparent discrepancy between the two measures. Gastrointestinal involvement also appeared more frequent among females, with only 20.9% of female patients exhibiting no gastrointestinal symptoms compared to 40% of males, a difference approaching statistical significance (*p* = 0.054). Additionally, peripheral vascular involvement tended to be more severe in females: 50.3% of female patients demonstrated severe manifestations in this domain compared to 30% of males (*p* = 0.085). Although renal involvement was more commonly observed in female patients, the difference did not reach statistical significance (*p* = 0.753). A comprehensive summary of differences in the severity of organ involvement by sex is provided in Table 2.

In a separate univariable logistic regression analysis with sex as the dependent variable, 2 clinical domains of the Medsger severity scale showed statistically significant associations. Grade 4 skin involvement was significantly associated with male sex (OR: 0.286; 95% CI: 1.014–18.115; *p* < 0.048), indicating that severe cutaneous involvement was more frequently observed in male patients. Similarly, the absence of lung involvement (grade 0) was also significantly associated with male sex (OR: 4.233; 95% CI: 1.361–13.163; *p* < 0.013), suggesting that male sex was associated with a lower frequency of lung involvement. Due to the small number of male patients in several Medsger severity score strata, multivariable analysis could not be performed reliably. As a result, we limited our evaluation to an exploratory univariable sex-based comparison to ensure robustness of the findings (Supplementary Table S2).

### 4.2. Correlation Analysis

Spearman’s rank correlation coefficient was used to evaluate associations between mRSS and selected continuous clinical variables. No significant correlations were found between mRSS and age (rho = 0.070, *p* = 0.334) or mean disease duration from NRP onset (rho = 0.044, *p* = 0.551). A borderline positive correlation was observed between mRSS and mean disease duration from RP onset (rho = 0.134, *p* = 0.067), as well as with PAPs measured in mmHg (rho = 0.122, *p* = 0.090) (Supplementary Table S3).

A strong correlation between the categorical Medsger severity skin domain and the continuous mRSS (r = 0.822, *p* < 0.001), indicating that both indices reflect a common underlying construct of skin involvement severity (Supplementary Table S4).

### 4.3. Univariable and Multivariable Linear Regression Analysis by mRSS

Given the significant association between male sex and severe skin involvement in the univariable logistic regression analysis (defined as a Medsger skin domain score of 4; *p* < 0.048) (see Supplementary Table S1), we further evaluated this relationship using linear regression analysis with the continuous mRSS score as a the outcome (Table 3), supported by a strong correlation between the Medsger skin domain and mRSS (r = 0.822, *p* < 0.001), indicating that both measures reliably reflect skin involvement severity (see Supplementary Table S1). However, in both univariable and multivariable linear regression models, sex was not significantly associated with mRSS (*p* = 0.345 for univariable and *p* = 0.206 for multivariable analysis) (Table 3). These findings suggest that while male patients are more likely to reach the most severe categorical level of skin involvement, sex does not independently predict the overall extent of skin fibrosis when assessed on a continuous scale. In the multivariable model adjusting for key confounders, ATA positivity (Coeff. b = 7.067, *p* = 0.001), active disease status (Coeff. b = 5.345, *p* = 0.000), and presence of ulcers (Coeff. b = 3.744, *p* = 0.024) remained independently associated with higher mRSS scores (Table 3).

## 5. Discussion

To our knowledge, this is the first multicenter study investigating sex differences in Egyptian SSc patients, revealing some differences in disease manifestations. Specifically, male patients exhibited more severe skin involvement as assessed by the Medsger’s severity scale and were more frequently treated with corticosteroids and PDEi. In contrast, female patients showed more frequent general systemic involvement according to the Medsger’s severity scale and had lower rates of lung involvement. Despite these findings, no other significant sex-based variations were observed, suggesting that in this cohort, sex alone may not represent a major determinant of disease severity in this population.

As expected, we confirmed the well-established female predominance in SSc, in line with previous studies [6,7,16], while no significant sex differences were observed in age at disease onset, clinical subtype, autoantibody profile, or major organ involvement. However, our data diverge from large-scale European Scleroderma Trials and Research Group (EUSTAR), which reported a higher prevalence of dcSSc and more severe organ involvement among male patients, even in those with lcSSc [17]. In contrast, within the Egyptian cohort, the proportion of male patients with lcSSc slightly exceeded that of dcSSc, although this difference did not reach statistical significance. These discrepancies may reflect geographic or genetic variability, environmental exposures, or differences in sample composition.

Autoantibody analysis showed uniformly high ANA positivity in both sexes, with no significant differences in ACA or ATA prevalence. This contrasts with previous reports describing higher ACA rates in female patients and increased ATA or anti-RNA Pol III in male patients [7,18]. The absence of RNA Pol III testing in our real-world cohort represents a limitation. Moreover, as up to 10% of “seronegative” patients may carry other, novel SSc-specific antibodies [19], future studies employing broader autoantibody panels are warranted.

Concerning systemic organ involvement, female patients more frequently had PAPs > 35 mmHg and higher Medsger’s lung scores, although neither difference reached statistical significance. These findings, consistent with previous studies [7,16], suggest a trend toward more pronounced pulmonary involvement in females. Gastrointestinal involvement also appeared more common in female patients, particularly esophageal dysmotility, whereas asymptomatic presentations were more frequent among males, consistent with previous Egyptian reports [20]. Moderate general systemic involvement was significantly more frequent in female patients (*p* = 0.010), whereas severe systemic involvement remained uncommon in both sexes. Kidney involvement was slightly more frequent in females but did not reach significance, differing from Western cohorts that report a higher male risk for renal crisis [18]. Regarding treatment, corticosteroid use was significantly higher among male patients (*p* = 0.027), with a trend toward greater PDE5i use, likely reflecting individualized treatment decisions rather than sex-driven severity. Overall, treatment approaches were broadly comparable between sexes.

Both sexes showed high rates of significant skin involvement (mRSS ≥ 14: 79.8% of females vs. 73.7% of males). However, severe skin damage was more frequent in males (Medsger skin score 4: 15.8% vs. 4.5%; *p* = 0.044), supporting a more aggressive male cutaneous phenotype [16]. Univariable analysis indicated that males were more likely to develop severe skin involvement although this association may reflect the small number of male patients and should be confirmed in larger cohorts. Importantly, no significant sex differences were observed in continuous mRSS values in either univariable or multivariable models, suggesting that sex alone does not independently predict the extent of skin fibrosis. The strong correlation between Medsger’s categorical scores and mRSS (Supplementary Table S1) supports the complementary use of both tools, as categorical scoring may capture subgroups not identified by continuous measures. Finally, multivariable analysis showed that ATA positivity, higher disease activity (EScSG score), and the presence of digital ulcers were independent predictors of higher mRSS values, underscoring the greater contribution of immunologic and vascular factors over sex in determining skin fibrosis severity.

This study has several limitations. Its cross-sectional design limits causal inference, and the absence of follow-up data prevents assessment of disease progression. The small number of male patients reduced statistical power and limited the ability to perform multivariable analysis to fully adjust for confounders. Autoantibody testing did not include key specificities such as anti-RNA polymerase III, and pulmonary arterial hypertension may have been underestimated due to the lack of right heart catheterization. Moreover, socio-cultural and economic factors were not evaluated, and treatment was not randomized, introducing potential confounding. Despite these limitations, this is the first multicenter study to investigate sex differences in SSc in an Egyptian cohort, addressing a regional knowledge gap and providing novel insights from a population largely underrepresented in global SSc research. By applying validated organ-specific severity indices, and both univariable and multivariable linear regression models with mRSS as a continuous outcome, we were able to improve the statistical power and account for potential confounders, despite the limited number of male patients [21]. This approach allowed for a more refined assessment of sex-specific differences in skin involvement and reinforced the conclusion that sex alone does not independently predict disease severity in this cohort.

## 6. Conclusions

In this Egyptian multicenter SSc cohort, sex had a limited impact on clinical heterogeneity. Other factors, genetic, environmental, immunological, and healthcare-related factors, likely play a more dominant role. Although some sex-related differences in organ involvement and treatment were observed, overall disease manifestations were comparable between sexes. Our exploratory analyses provide valuable insights for future, larger, and possibly multicenter longitudinal studies, particularly in male SSc patients, where the male sex is markedly underrepresented in the current literature. Furthermore, these investigations should also be extended to other connective tissue diseases in order to explore potential sex-related differences and to better define both shared mechanisms and disease-specific features across autoimmune disorders, especially in non-Western populations.

## Figures and Tables

**Table 1:** Comparison of demographic and disease-related features of SSc patients by sex.

Feature, n (%)	FEMALES (n = 177)	MALES (n = 20)	*p* Value
Age (mean ± SD, years)	41.2 ± 12.5	39.05 ± 13.07	0.424
LcSSc	106 (59.9)	14 (70)	0.380
DcSSc	71 (40.1)	6 (30)	0.380
Mean disease duration ±SD from non-RP onset, years	7.51 ± 6.09	5.56 ± 2.99	0.182
ANA positive	93	91.3	>0.05
ACA positive	62 (36.9)	6 (30)	0.544
ATA positive	54 (32.3)	5 (25)	0.505
mRSS ≥ 14	138 (79.8)	14 (73.7)	0.535
sPAP > 35 mmHg (echocardiogram)	67 (38.7)	5 (26.3)	0.289
ILD	106 (65.8)	9 (52.9)	0.290
EScSG (active disease)	97 (54.8)	8 (40)	0.209
CYC	18 (10.2)	1 (5)	0.458
MMF	23 (13)	4 (20)	0.388
AZA	27 (15.3)	5 (25)	0.263
MTX	97 (54.8)	8 (40)	0.209
HCQ	40 (22.6)	7 (35)	0.217
GC	54 (30.5)	11 (55)	0.027
CCB	156 (88.1)	18 (90)	0.806
PDEi (sildenafil)	18 (10.2)	5 (25)	0.050

ANA: antinuclear antibodies; ACA: anticentromere antibodies; ATA: antitopoisomerase I antibodies; mRSS: modified Rodnan skin score; ILD: interstitial lung disease; EScSG: European Scleroderma Study Group; lcSSc: limited cutaneous systemic sclerosis; dcSSc: diffuse cutaneous systemic sclerosis; CYC; cyclophosphamide; MMF: mycophenolate mofetil; AZA: azathioprine; MTX: methotrexate; HCQ: hydroxychloroquine; RTX: rituximab; TCZ: tocilizumab; CCB: calcium channels blockers; PDEi: phosphodiesterase inhibitors; ACEi: angiotensin converting enzyme inhibitors.

**Table 2:** Differences in the Severity of Organ Involvement by sex.

Feature, n (%)	FEMALES (n = 177)	MALES (n = 20)	*p* Value
General			
0	42 (24.4)	6 (30)	0.585
1	83 (48.3)	14 (70)	0.066
2	44 (25.6)	0 (0)	0.010
3	3 (1.7)	0 (0)	0.552
4	0 (0)	0 (0)	-
Peripheral vascular			
0	13 (7.3)	2 (10)	0.671
1	38 (21.5)	5 (25)	0.717
2	37 (20.9)	7 (35)	0.151
3	89 (50.3)	6 (30)	0.085
4	0 (0)	0 (0)	-
Skin			
0	7 (4)	1 (5)	0.822
1	40 (22.7)	4 (21.1)	0.868
2	79 (44.9)	10 (52.6)	0.520
3	42 (23.9)	1 (5.3)	0.063
4	8 (4.5)	3 (15.8)	0.044
Joint/tendon			
0	76 (43.2)	8 (40)	0.785
1	62 (35.2)	7 (35)	0.984
2	34 (19.3)	5 (25)	0.546
3	4 (2.3)	0 (0)	0.496
4	0 (0)	0 (0)	-
Muscles			
0	76 (42.9)	8 (40)	0.801
1	85 (48)	10 (50)	0.867
2	14 (7.9)	2 (10)	0.746
3	2 (1.1)	0 (0)	0.633
4	0 (0)	0 (0)	-
GI			
0	37 (20.9)	8 (40)	0.054
1	86 (48.6)	6 (30)	0.114
2	48 (27.1)	6 (30)	0.784
3	2 (1.1)	0 (0)	0.633
4	4 (2.3)	0 (0)	0.497
Heart			
0	39 (22)	2 (10)	0.209
1	73 (41.2)	8 (40)	0.915
2	49 (27.7)	8 (40)	0.250
3	16 (9)	2 (10)	0.888
4	0 (0)	0 (0)	-
Lung			
0	86 (48.6)	16 (80)	0.008
1	67 (37.9)	4 (20)	0.115
2	22 (12.4)	0 (0)	0.094
3	2 (1.1)	0 (0)	0.633
4	0 (0)	0 (0)	-
Kidney			
0	155 (87.6)	18 (90)	0.753
1	11 (6.2)	1 (5)	0.830
2	7 (4)	1 (5)	0.822
3	4 (2.3)	0 (0)	0.497
4	0 (0)	0 (0)	-

GI: gastrointestinal tract; 0: normal; 1: mild; 2: moderate; 3 severe; 4: end stage.

**Table 3:** Univariable and multivariable linear regression (mRSS as a dependent continuous variable).

Variables	Coeff. b	95% CI	*p* Value
**Univariable Analysis**			
Gender (male)	0.666	−4.381 to 5.713	0.345
ACA positive	−1.523	−4.653 to 1.606	0.234
ATA positive	9.206	6.236 to 12.177	0.000
NON ACA/ATA	−4.364	−7.978 to −0.750	0.018
ILD	7.430	4.175 to 10.685	0.000
PAPs > 35 mmHg	1.563	0.759 to 6.925	0.015
EScSG (active)	8.021	5.242 to 10.799	0.000
Ulcers	8.262	5.149 to 11.375	0.000
Multivariable Analysis			
Gender (male)	2.875	0.206 to −1.593	0.206
Mean disease duration from RP onset	0.139	−0.080 to 0.362	0.220
ATA positive	7.067	4.070 to 10.064	0.000
EScSG (active)	5.345	2.450 to −8.240	0.000
Ulcers	3.744	0.503 to 6.985	0.024

ACA: anticentromere antibody; ATA; anti-topoisomerase I antibody; NON ACA/ATA: double negative for anticentromere and anti-topoisomerase I antibody; ILD: interstitial lung disease; EScSG: European Scleroderma Study Group.

## Data Availability

The data presented in this study are available on request from the corresponding author. The data are not publicly available due to privacy and ethical restrictions.

## Supplementary Materials

The following supporting information can be downloaded at: https://www.mdpi.com/article/10.3390/jcm14217574/s1, Table S1. Sex-Matched Sub-Analysis According to Disease Severity and age; Table S2. Univariable logistic regression analyses of Medsger’s Severity Scale by sex; Table S3. Correlation analysis (mRSS as a dependent variable); Table S4. Correlation analysis between mRSS (dependent continuous variable) and Medsger severity skin score.

## Abbreviations

The following abbreviations are used in this manuscript:

ANA	antinuclear antibodies
ACA	anticentromere antibodies
ACEi	angiotensin converting enzyme inhibitors
ATA	antitopoisomerase I antibodies
AZA	azathioprine
CCB	calcium channels blockers
CYC	cyclophosphamide
DcSSc	diffuse cutaneous systemic sclerosis
EScSG	European Scleroderma Study Group
GI	gastrointestinal tract
HCQ	hydroxychlorochine
ILD	interstitial lung disease
LcSSc	limited cutenaous systemic sclerosis
MMF	mycophenolate mofetile
MTX	methotrexate
PDEi	phosphodiesterase inhibitors
RTX	rituximab
TCZ	tocilizumab
