# Supplementary material for: Sex-Based Differences in Systemic Sclerosis Among Egyptian Patients: Insights from a Multicenter Observational Study in a Genetically Distinct North African Mediterranean Population
Source: J Clin Med. 2025 Oct 25;14(21):7574. doi: 10.3390/jcm14217574 (PMC12609230; doi:10.3390/jcm14217574)
Supplement: Supplementary file 1 [file jcm-14-07574-s001.zip › jcm-3866676-supplementary.pdf]

Supplementary Tables

Supplementary Table S1. Sex-Matched Sub-Analysis According to Disease Severity and age

Feature, n (%)	FEMALES (n =20)	MALES (n =20)	P value
Age (mean±SD, years)	41.2±12.5	39.05±13.07	0.424
LcSSc	10 (50)	14 (70)	0.197
DcSSc	10 (50)	6 (30)	0.197
Mean disease duration ±SD from non-RP onset, years	7.51±6.09	5.56±2.99	0.182
ANA positive	93%	91.3	>0.05
ACA positive	4 (21.1)	6 (30)	0.522
ATA positive	6 (33.3)	5 (25)	0.572
ACA/ATA negative	6 (31.6)	6 (33.3)	0.909
mRSS≥14	17 (85)	14 (73.7)	0.382
sPAP>35 mmHg (echocardiogram)	8 (42.1)	5 (26.3)	0.305
ILD	9 (64.3)	9 (52.9)	0.524
Ulcers (past/current)	14 (70)	13 (65)	0.736
EScSG (active disease)	12 (60)	8 (40)	0.206
Medsger's severity scale<br>General			
0	17 (85)	19 (95)	0.292
1	3 (15)	1 (5)	0.292
2	0 (0)	0 (0)	-
3	0 (0)	0 (0)	-
4	0 (0)	0 (0)	-
Peripheral vascular			
0	2 (10)	3 (15)	0.633
1	14 (70)	5 (60)	0.52
2	2 (10)	2 (10)	1.000
3	2 (10)	3 (15)	0.633
4	0 (0)	0 (0)	-
Skin			
0	9 (45)	11 (55)	0.527
1	11 (55)	7 (35)	0.204
2	0 (0)	2 (10)	0.147
3	0 (0)	0 (0)	-
4	0 (0)	0 (0)	-
Joint/tendon			
0	0 (0)	0 (0)	-
1	0 (0)	1 (5)	0.311

2	1 (5)	0 (0)	0.311
3	0 (0)	1 (5)	0.311
4	1 (5)	0 (0)	0.311
Muscles			
0	20 (100)	18 (90)	0.147
1	0 (0)	1 (5)	0.311
2	0 (0)	0 (0)	-
3	0 (0)	0 (0)	-
4	0 (0)	1 (5)	0.311
GI			
0	8 (42.1)	10 (50)	0.621
1	11 (57.9)	10 (50)	0.621
2	0 (0)	0 (0)	-
3	0 (0)	0 (0)	-
4	0 (0)	0 (0)	-
Heart			
0	7 (35)	3 (15)	0.144
1	5 (25)	3 (15)	0.419
2	6 (30)	8 (40)	0.507
3	2 (10)	6 (30)	0.114
4	0 (0)	0 (0)	-
Lung			
0	17 (85)	17 (85)	1.000
1	2 (10)	1 (5)	0.548
2	1 (5)	1 (5)	1.000
3	0 (0)	1 (5)	0.311
4	0(0)	0 (0)	-
Kidney			
0	19 (95)	19 (95)	1.000
1	1 (5)	1 (5)	1.000
2	0 (0)	0 (0)	-
3	0 (0)	0 (0)	-
4	0(0)	0 (0)	-

ANA: antinuclear antibodies; ACA: anticentromere antibodies; ATA: antitopoisomerase I antibodies; mRSS: modified Rodnan skin score; ILD: interstitial lung disease; EScSG: European Scleroderma Study Group; lcSSc: limited cutenaous systemic sclerosis; dcSSc: diffuse cutaneous systemic sclerosis; GI: gastrointestinal involvement

Supplementary Table S2. Univariable logistic regression analyses of Medsger's Severity Scale by sex

Univariate analyses	OR	SE	*P*	CI 95%
**General**				
0	1.327	0.519	0.586	0.479-3.670
1	2.502	0.511	0.073	0.919-6.815
2	-	-	-	-
3	-	-	-	-
4	-	-	-	-
Peripheral vascular				
0	1.402	0.799	0.673	0.293-6.712
1	1.219	0.548	0.717	0.417-3.568
2	2.037	0.504	0.158	0.759-5.471
3	0.424	0.511	0.093	0.156-1.153
4	-	-	-	-
Skin				
0	0.879	1.076	0.905	0.107-7.248
1	1.378	0.519	0.537	0.498-3.809
2	0.992	0.474	0.987	0.392-2.513
3	0.193	1.042	0.114	0.025-1.485
4	4.286	0.735	**0.048**	1.014-18.115
Joint/tendon				
0	0.785	0.481	0.785	0.342-2.252
1	0.990	0.495	0.984	0.376-2.610
2	1.392	0.551	0.548	0.473-4.096
3	-	-	-	-
4	-	-	-	-
Muscle				
0	0.886	0.481	0.801	0.345-2.274
1	1.082	0.472	0.867	0.429-2.729
2	1.294	0.796	0.746	0.272-6.153
3	-	-	-	-
4	-	-	-	-
GI				
0	2.523	0.492	0.060	0.961-6.622
1	0.453	0.511	0.121	0.167-1.234
2	1.152	0.075	0.784	0.419-3.169
3	-	-	-	-
4	-	-	-	-
Heart				
0	0.393	0.767	0.224	0.087-1.768
1	0.950	0.481	0.915	0.370-2.439
2	1.741	0.486	0.254	0.671-4.518
3	1.118	0.790	0.888	0.238-5.260
4	-	-	-	-
Lung				
0	4.233	0.579	**0.013**	1.361-13.163
1	0.410	0.580	0.125	0.132-1.279
2	-	-	-	-
3	-	-	-	-
4	-	-	-	-
Kidney				
0	1.277	0.779	0.753	0.277-5.885
1	0.794	1.072	0.830	0.097-6.495
2	1.278	1.096	0.823	0.149-10.954
3	-	-	-	-
4	-	-	-	-

GI: gastrointestinal tract; 0: normal; 1: mild; 2: moderate; 3 severe; 4: endstage

Supplementary Table S3. Correlation analysis (mRSS as a dependent variable).

Correlates	N	Spearman Rank Correlation Coefficient	P value
**mRSS as a dependent variable**			
Age	195	0.070	0.334
Mean disease duration from NRP onset	188	0.044	0.551
PAPs mmHg (continuous)	195	0.122	0.090

mRSS: modified Rodnan skin score; NRP; symptom non-Raynaud's phenomenon; RP: Raynaud's phenomenon; PAPs: pulmonary arterial pressure estimated by echocardiography

Supplementary Table S4. Correlation analysis between mRSS (dependent continuous variable) and Medsger severity skin score.

Correlates	N	Spearman Rank Correlation Coefficient	P value
**mRSS as a dependent continuous variable**			
Medsger severity skin score	195	0.822	**0.000**

mRSS: modified Rodnan skin score
